# Supplementary material for: Planning Strategy to Optimize the Dose-Averaged LET Distribution in Large Pelvic Sarcomas/Chordomas Treated with Carbon-Ion Radiotherapy
Source: Cancers (Basel). 2023 Oct 9;15(19):4903. doi: 10.3390/cancers15194903 (PMC10571585; doi:10.3390/cancers15194903)
Supplement: Supplementary file 1 [file cancers-15-04903-s001.zip › Supplementary Figure S1.pdf]

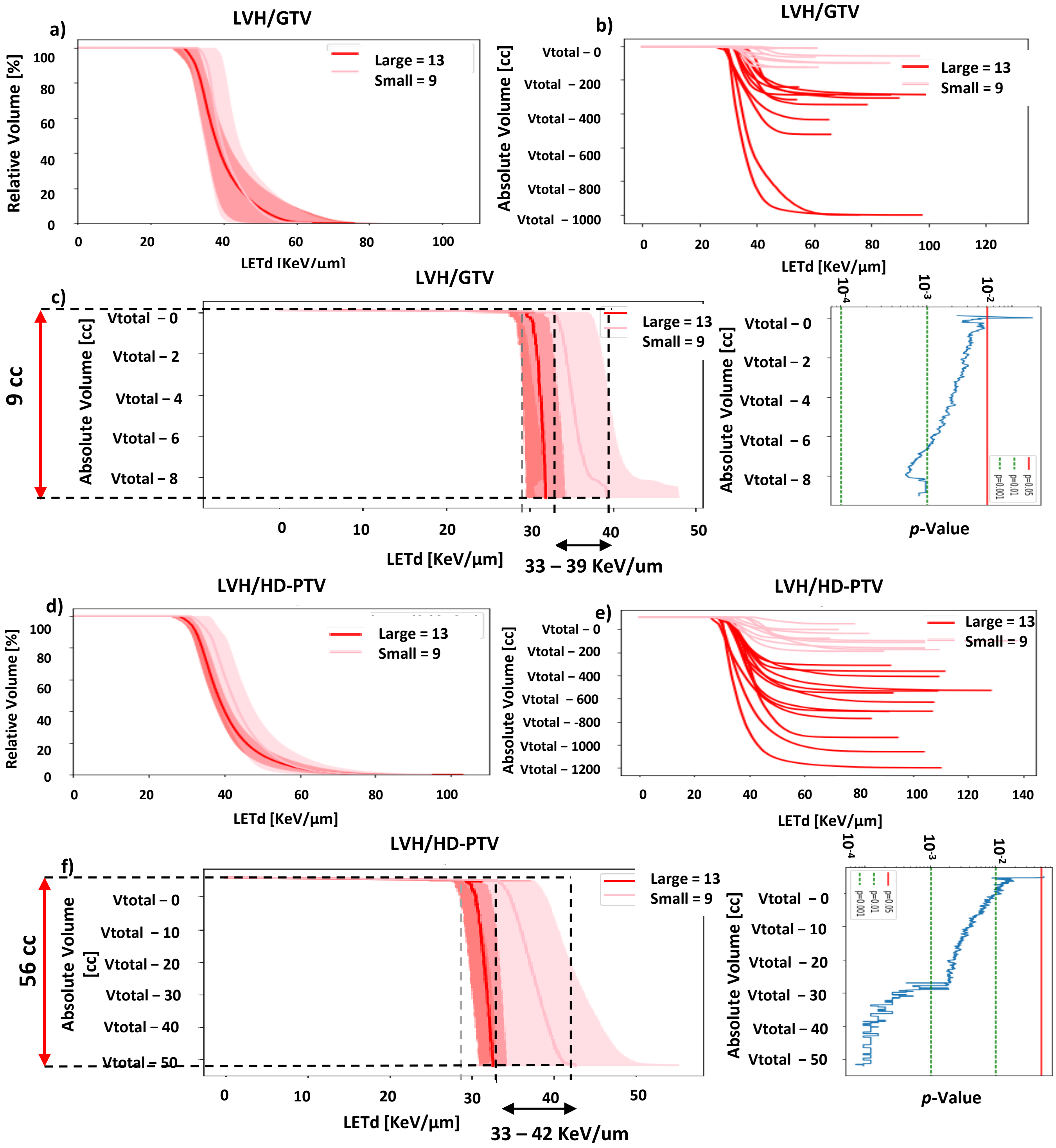

S1. LVH Evaluation for GTV and HD-PTV in small and large pelvic sarcomas/chordomas A relative-volume cumulative LVH for (a) GTV and (d) HD-PTV of small and large pelvic sarcoma (mean ± SD), with LVH plot showing difference in LETd distribution. An absolute-volume LVH plots of individual cases shifted with respect to largest (b) GTV and (e) HD-PTV (Vtotal = volume of largest HD-PTV/GTV), demonstrating distinct separation between LVH plots of large and small tumors. An absolute-volume cumulative LVH for (c) GTV and (f) HD-PTV of small and large pelvic sarcoma (mean ± SD), showing a statistically significant difference ($p<0.05$) between interval of max tumor volume of at least 56 cm^3, for HD-PTV and 9 cm^3, for GTV that should receive LETd of at least ≥33-42 keV/µm.
