# Supplementary material for: Planning Strategy to Optimize the Dose-Averaged LET Distribution in Large Pelvic Sarcomas/Chordomas Treated with Carbon-Ion Radiotherapy
Source: Cancers (Basel). 2023 Oct 9;15(19):4903. doi: 10.3390/cancers15194903 (PMC10571585; doi:10.3390/cancers15194903)
Supplement: Supplementary file 1 [file cancers-15-04903-s001.zip › Supplementary Figure S2.pdf]

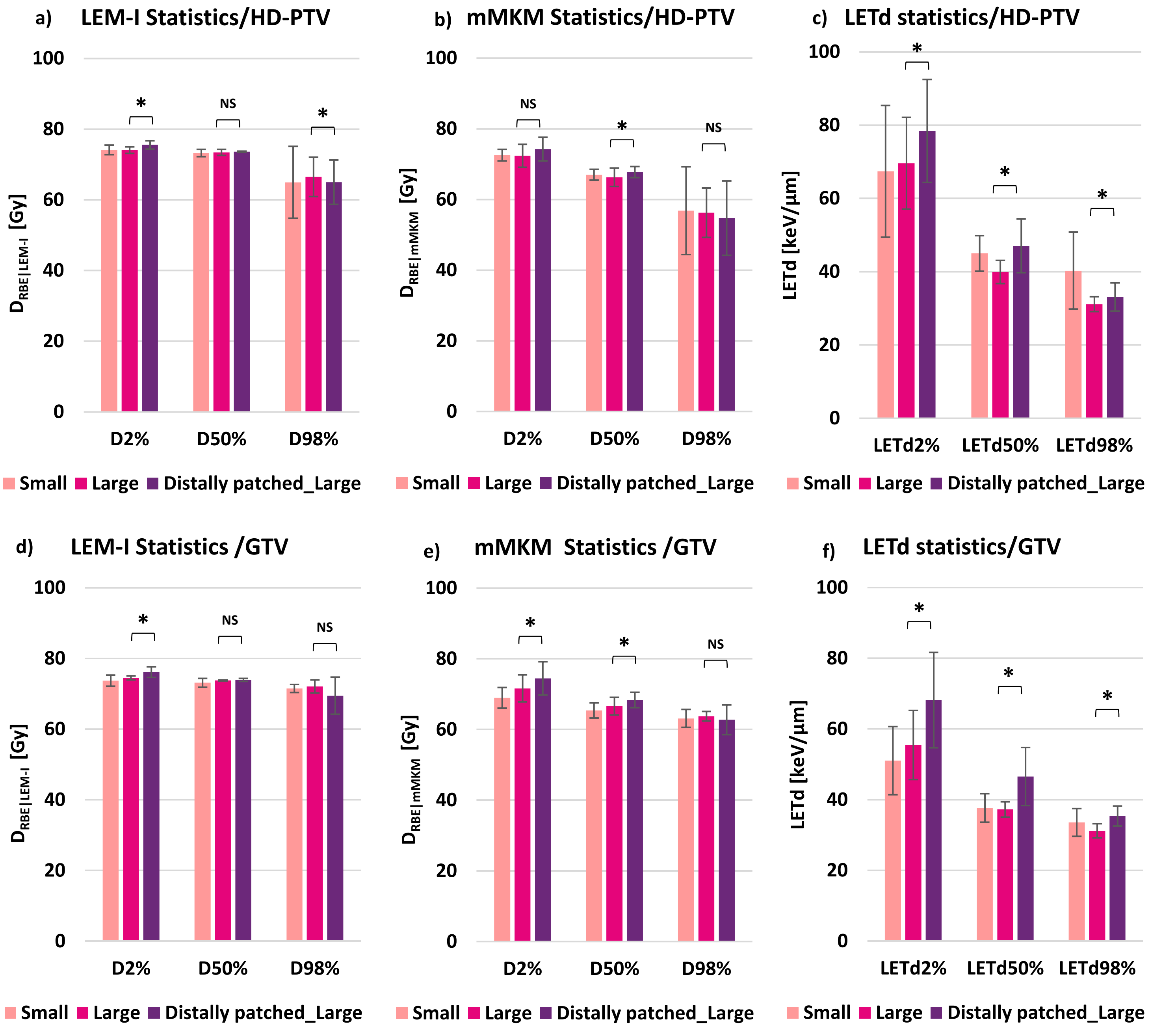


Figure S2. LETd optimization using 'distal-patching': $D_{RBE|LEM-I}$, $D_{RBE|mMKM}$ and LETd statistics for GTV and HD-PTV. a–c) $D_{RBE}$ parameters D2%, D50%, D98% for LEM-I and for mMKM, LETd statistics for small tumor, large tumors with un-patched plans and distally-patched plans for HD-PTV. (d–f) DRBE parameters D2%, D50%, D98% for LEM-I and for mMKM, LETd statistics for small tumor, large tumors with unpatched plans and distally-patched plans for GTV. (n = 22, small = 9, Large unpatched = 13, Large-distally patched). *: p <0.05, NS: p = not significant.
