# Supplementary material for: Planning Strategy to Optimize the Dose-Averaged LET Distribution in Large Pelvic Sarcomas/Chordomas Treated with Carbon-Ion Radiotherapy
Source: Cancers (Basel). 2023 Oct 9;15(19):4903. doi: 10.3390/cancers15194903 (PMC10571585; doi:10.3390/cancers15194903)
Supplement: Supplementary file 1 [file cancers-15-04903-s001.zip › Supplementary Figure S3.pdf]

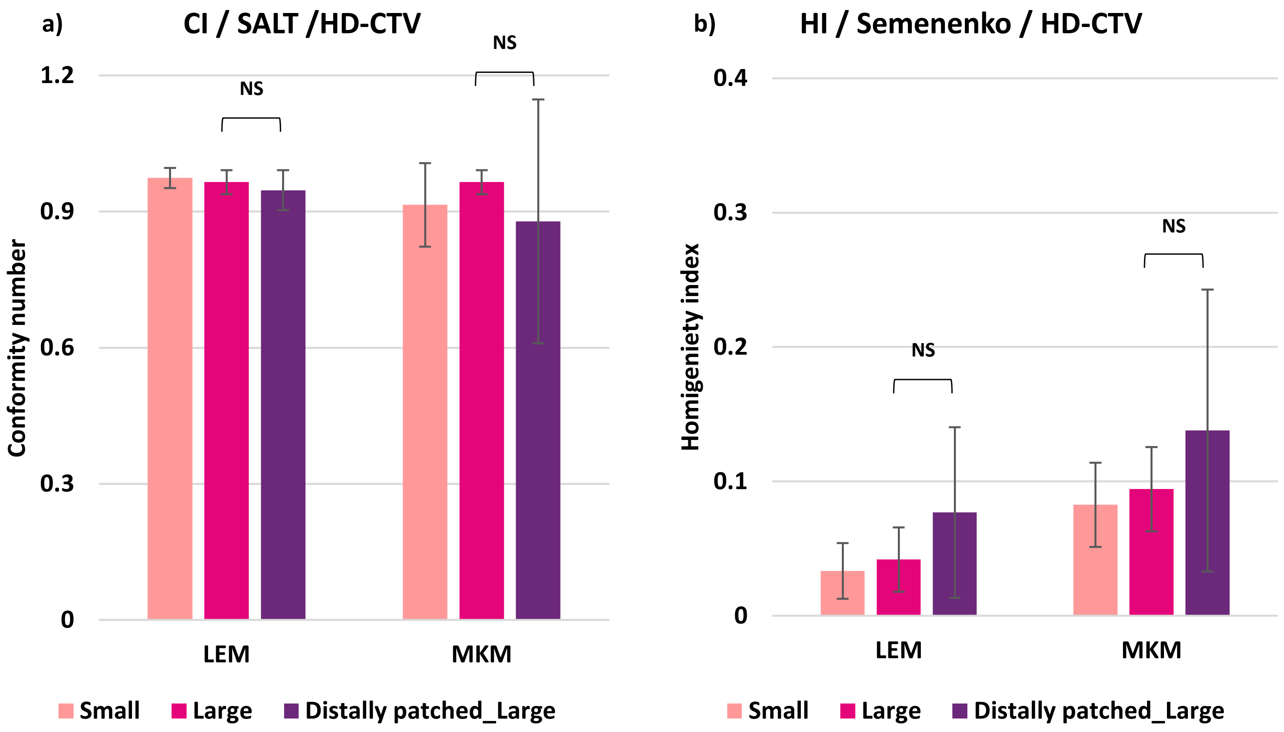

Figure S3. (a) Conformity index (left panel) and (b) homogeneity index (right panel) for small tumor, large tumors with unpatched plans and distally-patched plans. (n = 22, small = 9, Large unpatched = 13, Large- distally patched = 13).
