# Supplementary material for: Planning Strategy to Optimize the Dose-Averaged LET Distribution in Large Pelvic Sarcomas/Chordomas Treated with Carbon-Ion Radiotherapy
Source: Cancers (Basel). 2023 Oct 9;15(19):4903. doi: 10.3390/cancers15194903 (PMC10571585; doi:10.3390/cancers15194903)
Supplement: Supplementary file 1 [file cancers-15-04903-s001.zip › Supplementary Figure S4.pdf]

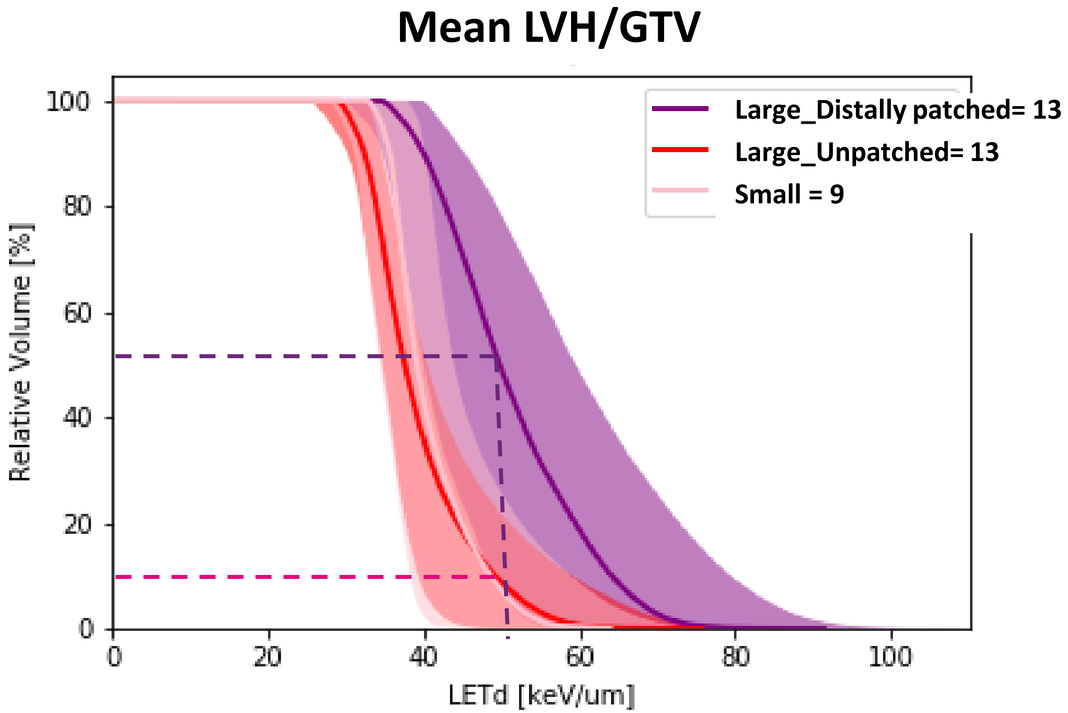

Figure S4. Cumulative relative-volume histogram (mean ± SD) showing the fraction of GTV receiving >50 keV/µm improved from <10% in unpatched plans to ≥50% in distally-patched plans in large tumors. .
