# Supplementary material for: Planning Strategy to Optimize the Dose-Averaged LET Distribution in Large Pelvic Sarcomas/Chordomas Treated with Carbon-Ion Radiotherapy
Source: Cancers (Basel). 2023 Oct 9;15(19):4903. doi: 10.3390/cancers15194903 (PMC10571585; doi:10.3390/cancers15194903)
Supplement: Supplementary file 1 [file cancers-15-04903-s001.zip › Supplementary Figure S5.pdf]

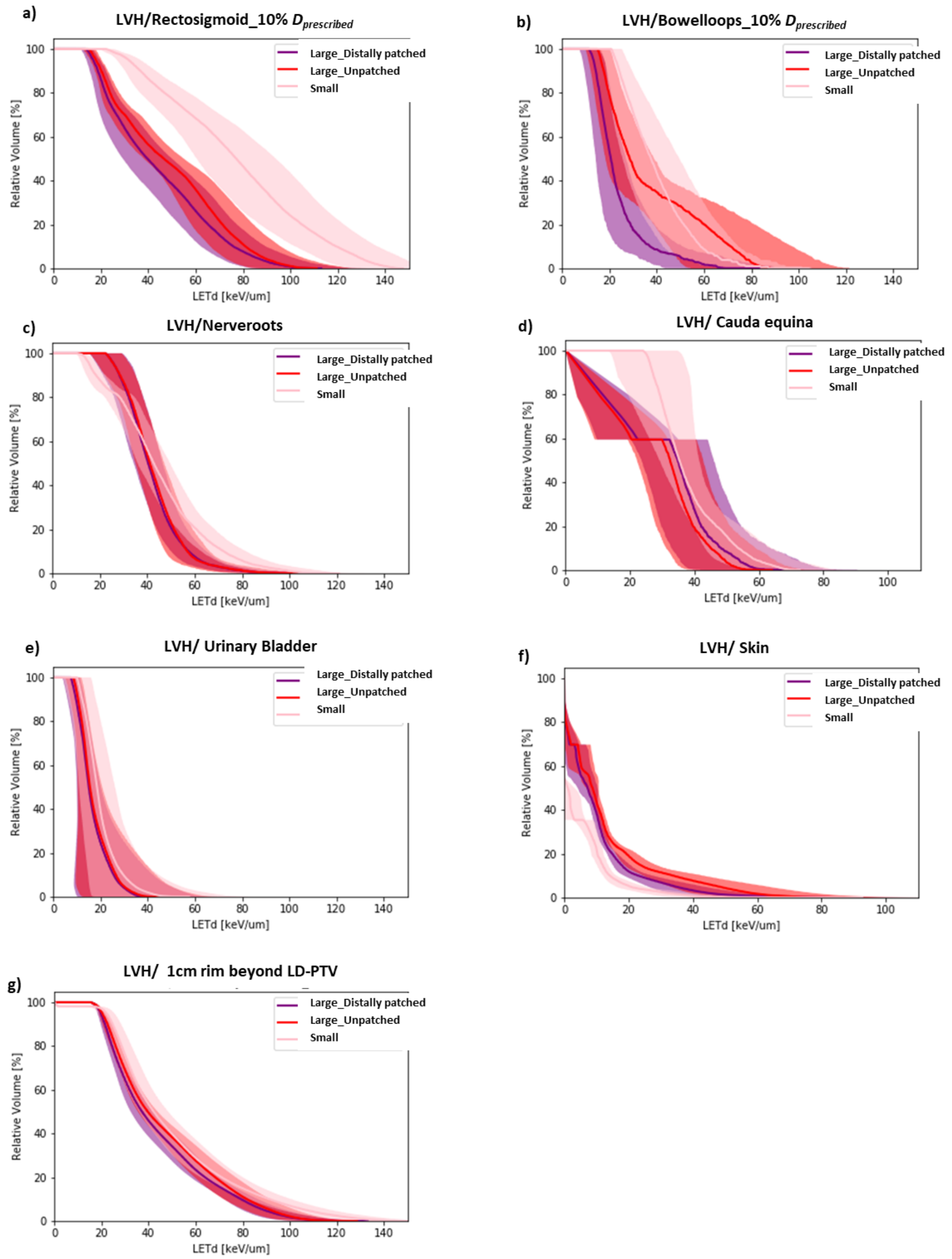

Figure S5. LETd optimization 'distal-patching': Cumulative relative-volume LVH profiles (mean ± SD) for OARs (a) Rectosigmoid filtered for <10% Dprescribed, (b) Small intestine/bowelloops filtered for <10% Dprescribed, (c) sacral nerveroots, (d) cauda equina, (e) urinary bladder, (f) skin (g) 1 cm shell around LD-PTV showing acceptable LETd distribution beyond LD-PTV in distally-patched plans in large tumors compared to unpatched plans in large and small tumors. (Note less sample size in 2 organs due to filtering out low doses. Rectosigmoid filtered for <10% Dprescribed: small: 9, Large distally-patched: 11, Large unpatched:9, Small intestine/bowelloops filtered for <10% Dprescribed, small: 5, Large distally-patched: 6, Large unpatched:6).
