# Supplementary material for: Planning Strategy to Optimize the Dose-Averaged LET Distribution in Large Pelvic Sarcomas/Chordomas Treated with Carbon-Ion Radiotherapy
Source: Cancers (Basel). 2023 Oct 9;15(19):4903. doi: 10.3390/cancers15194903 (PMC10571585; doi:10.3390/cancers15194903)
Supplement: Supplementary file 1 [file cancers-15-04903-s001.zip › Supplementary Figure S6.pdf]

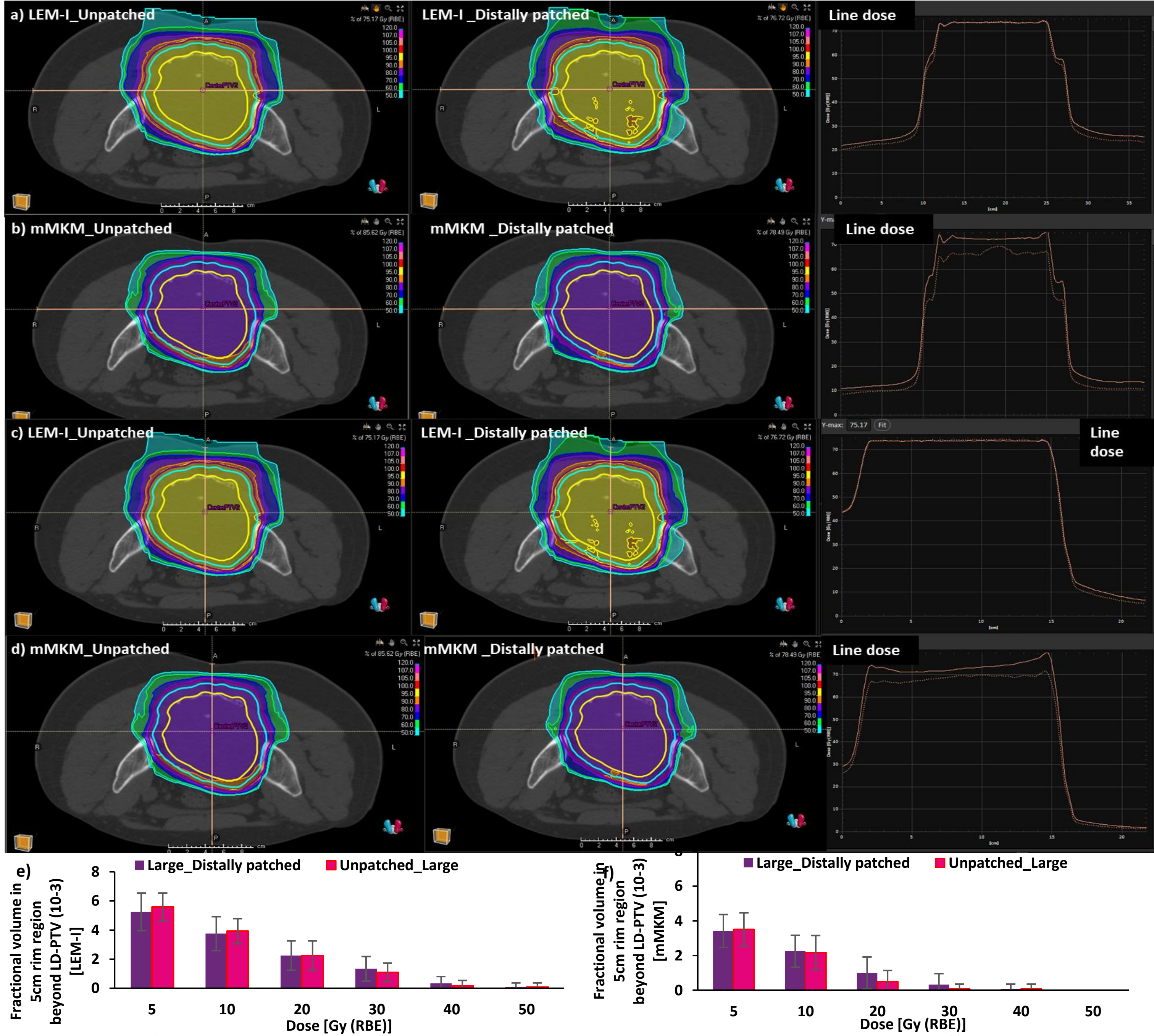

Figure S6. LETd optimization 'distal-patching': entrance region $D_{RBE|LEM-I}$, $D_{RBE|mMKM}$ evaluation. No difference in $D_{RBE|LEM-I}$, $D_{RBE|mMKM}$ in entrance dose region respectively is noted between distally-patched (dotted line) and unpatched (solid line) plan with respect to horizontal line doses. (a-b) with respect to vertical line doses (b-d). (e–f) Isodose levels 5, 10, 20, 30, 40 & 50 Gy (RBE) were converted into structures. There was no difference in volumes of isodose surfaces in the range of 5-50 Gy (RBE) between distally-patched and unpatched plans.
