# Supplementary material for: Planning Strategy to Optimize the Dose-Averaged LET Distribution in Large Pelvic Sarcomas/Chordomas Treated with Carbon-Ion Radiotherapy
Source: Cancers (Basel). 2023 Oct 9;15(19):4903. doi: 10.3390/cancers15194903 (PMC10571585; doi:10.3390/cancers15194903)
Supplement: Supplementary file 1 [file cancers-15-04903-s001.zip › Supplementary Figure S7.pdf]

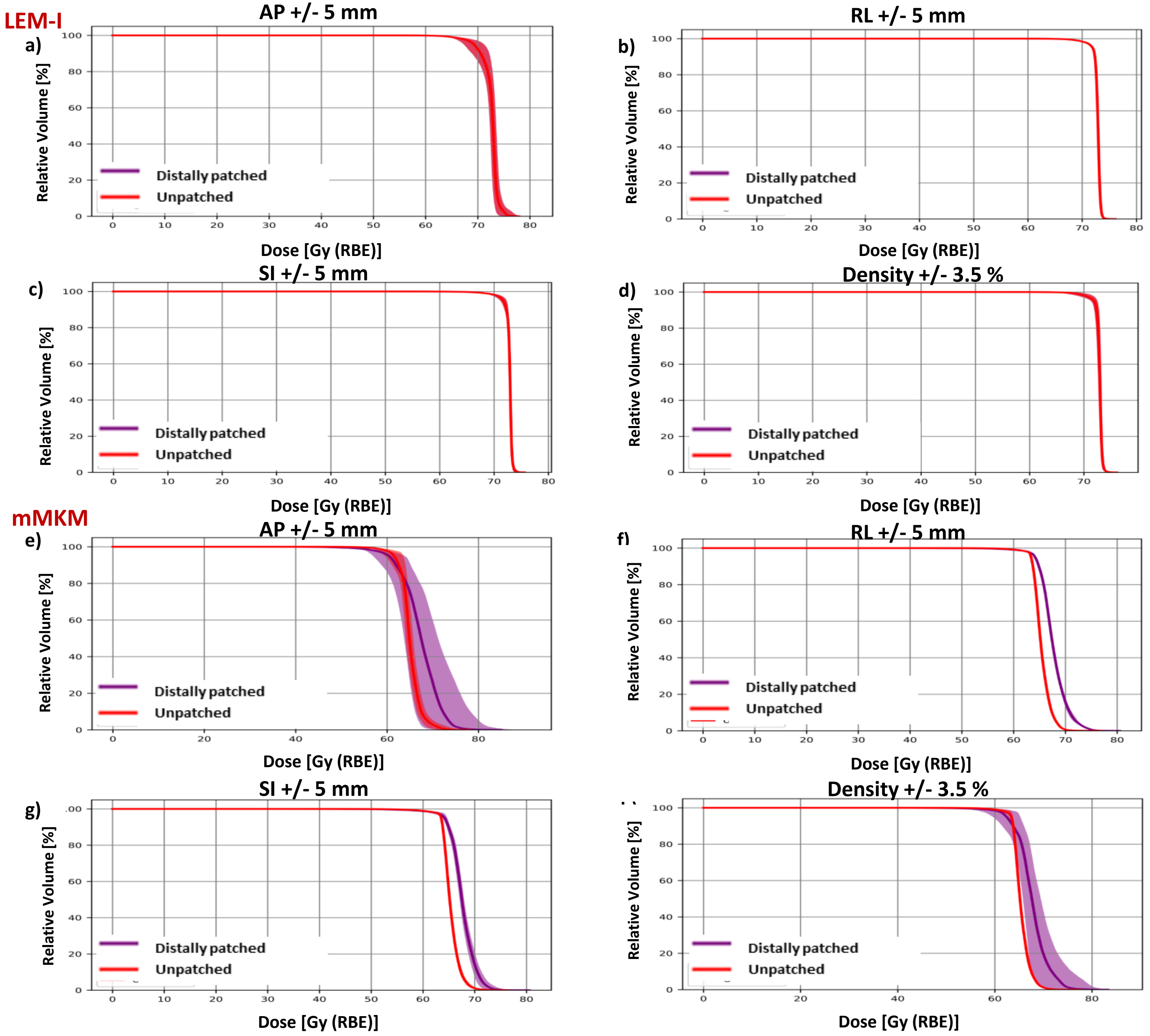

Figure S7. A representative case of large pelvic chordoma demonstrating an evaluation of robustness with respect to range (density ± 3.5%) and setup uncertainties [± 5mm shifts in antero-posterior (AP), supero-inferior (SI) and right and left (RL)] for $D_{RBE|LEM\text{-}I}$, $D_{RBE|mMKM}$ distribution. (a–d) Distally-patched plan shows compromise in LEM-I target coverage for HD-CTV for antero-posterior shifts. (e–h) Distally-patched plan shows slight compromise in mMKM target coverage for HD-CTV for antero-posterior shifts and range uncertainties.
