# Supplementary material for: Planning Strategy to Optimize the Dose-Averaged LET Distribution in Large Pelvic Sarcomas/Chordomas Treated with Carbon-Ion Radiotherapy
Source: Cancers (Basel). 2023 Oct 9;15(19):4903. doi: 10.3390/cancers15194903 (PMC10571585; doi:10.3390/cancers15194903)
Supplement: Supplementary file 1 [file cancers-15-04903-s001.zip › Supplementary Figure S9.pdf]

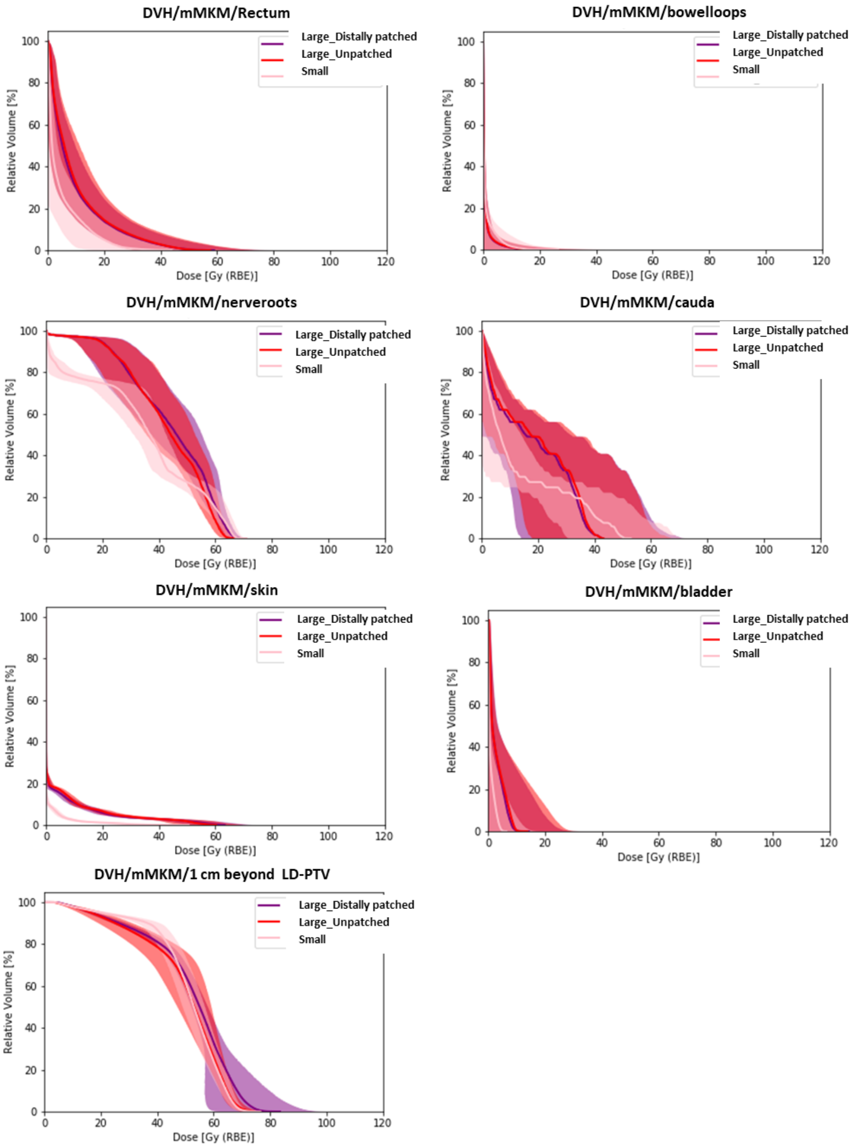

Figure S9. LETd optimization using ‘distal-patching’: Cumulative relative-volume DVH (mMKM) profiles (mean ± SD) for OARs (a) Rectum, (b) Small intestine/bowelloops, (c) sacral nerveroots, (d) cauda equina, (e) skin, (f) urinary bladder and g) 1 cm shell around LD-PTV showing no significant difference in mMKM distribution in distally-patched plans in large tumors compared to unpatched plans in large and small tumors.
